# Identification of *ANLN* as *ETV6* partner gene in recurrent t(7;12)(p15;p13): a possible role of deregulated *ANLN* expression in leukemogenesis

**DOI:** 10.1186/s12943-015-0471-5

**Published:** 2015-11-19

**Authors:** Paulo Vidal Campregher, Welbert de Oliveira Pereira, Bianca Lisboa, Renato Puga, Ricardo Helman, Mariana Miyagi, Evelyn Helena Ascendino da Mata, Tarcila Santos Datoguia, Elvira Deolinda Rodrigues Pereira Velloso, Nydia Strachman Bacal, Jeffrey S. Ross, Siraj Ali, Vincent Miller, Fernando Ferreira Costa, Nelson Hamerschlak, Fabio Pires de Souza Santos

**Affiliations:** Centro de Pesquisa Clinica, IIEP, Hopsital Albert Einstein, Av. Albert Einstein, 627/520, São Paulo, SP Brazil; Research Institute, Hospital Israelita Albert Einstein, São Paulo, Brazil; Department of Hematology, Hospital Israelita Albert Einstein, São Paulo, Brazil; Foundation Medicine, Cambridge, MT USA; Department of Pathology and Laboratory Medicine, Albany Medical College, Albany, NY USA; Department of Hematology, University of Campinas/Hemocentro - Unicamp, Campinas, Brazil

**Keywords:** Leukemia, Myeloid, Acute, Gene fusion, Translocation, Genetic

## Abstract

The *ETV6* gene encodes an ETS family transcription factor that is involved in a myriad of chromosomal rearrangements found in hematological malignancies and other neoplasms. A recurrent *ETV6* translocation, previously described in patients with acute myeloid leukemia (AML) (Genes Chromosomes Cancer 51:328–337,2012, Leuk Res 35:e212-214, 2011), whose partner has not been identified is t(7;12)(p15;p13). We herein report that the t(7;12)(p15;p13) fuses *ETV6* to *ANLN*, a gene not previously implicated in the pathogenesis of hematological malignancies, and we demonstrate that this translocation leads to high expression of the fusion transcript in the myeloid and lymphoid lineages.

## Findings

### Background

Balanced translocations leading to chimeric fusion genes play a major role in the pathogenesis of cancer. *ETV6* is a frequently rearranged gene involved in least 30 fusion genes in myeloid and lymphoid neoplasms [1]. A recurrent ETV6 translocation, previously described in patients with acute myeloid leukemia (AML), whose partner has not been identified is t(7;12)(p15;p13) [2, 3]. In the present work we describe the identification of ANLN as a novel *ETV6* fusion partner as a result of the t(7;12)(p15;p13).

### Results

**Fig. 1:**
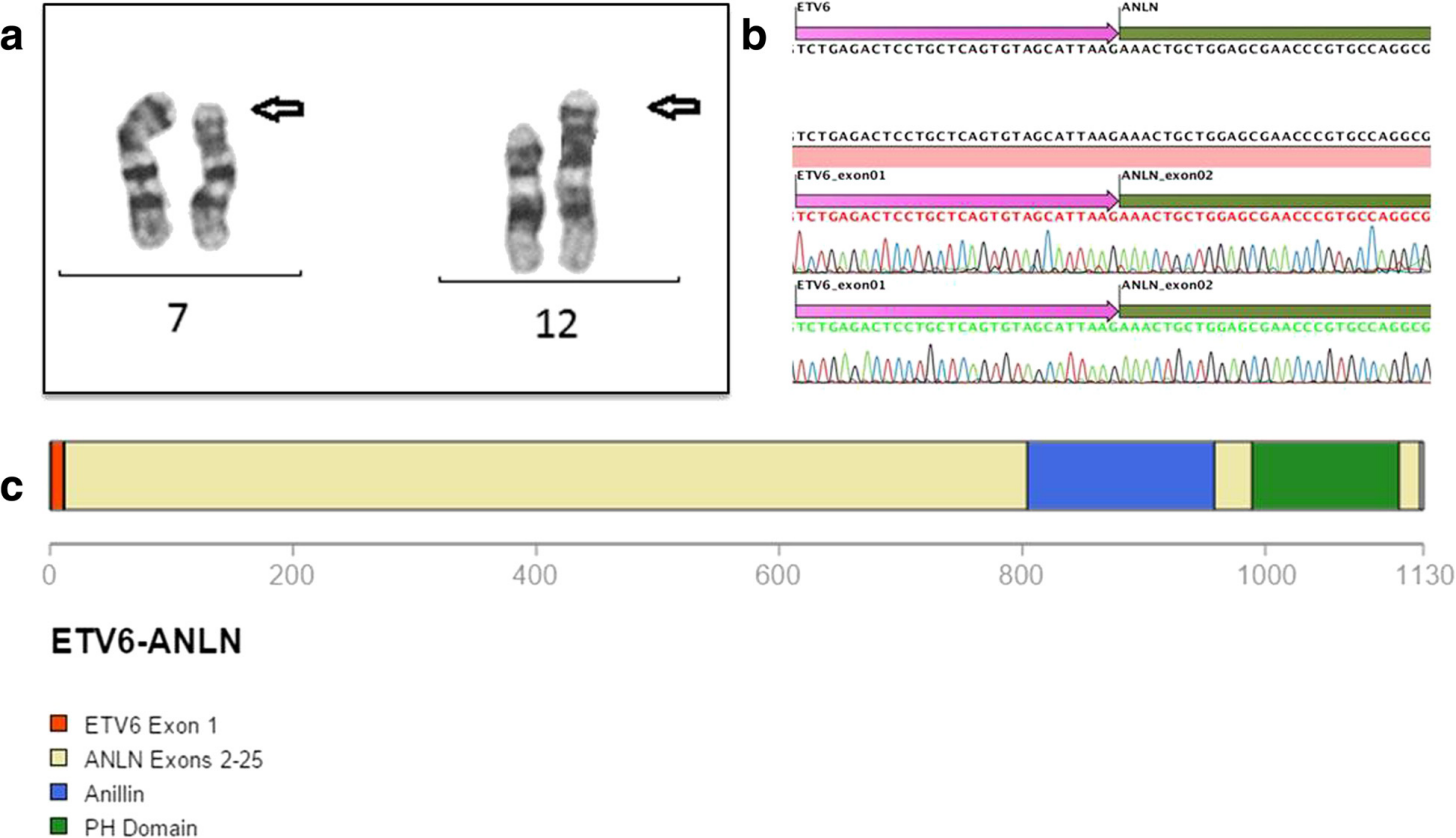
t(7;12)(p15;p13) and ETV6-ANLN fusion transcript. **a** Cytogenetic analysis revealing the t(7;12)(p15;p13) **b** Sanger sequencing of the fusion transcript, demonstrating the fusion of ETV6 exon 1 to ANLN exon 2. **c** Putative fusion protein highlighting ETV6 exon 1 and ANLN Anilin and PH domains

After Institutional Review Board approval (IRB-25000.179520/2011-36) and signing of consent form, we studied a 40 year-old female patient (P005) with diagnosis of *JAK2* V671F-positive primary myelofibrosis (PMF) and karyotype compatible with 46,XX,del(5)(q12q33) since 2010. She had been treated with supportive care and ruxolitinib. In 2013, the patient presented worsening of blood counts and increase in spleen size, suggesting disease progression. A bone marrow (BM) biopsy showed an increase in BM fibrosis (grade 3 out of 3) and 9 % blasts in the BM aspirate. Chromosomal analysis of BM cells showed 46,XX,del(5)(q12q33),t(7;12)(p15;p13) [20] (Fig. 1a). The patient was treated with an allogeneic BM transplantation from her HLA-haploidentical sister. Seven months after transplantation, she progressed to refractory acute megakaryoblastic leukemia that presented with two BM blast populations, a CD34-positive (4.9 %) and a CD34-negative (49.6 %).

At the time of disease progression before transplantation, a sample of her BM aspirate was analyzed by a hybrid-capture-based comprehensive genomic profiling assay (FoundationOne Heme), employing both DNAseq and RNAseq in a CLIA certified laboratory (Foundation Medicine, Cambridge, MA, USA). This method evaluates the entire coding sequence of 405 cancer-related genes, 31 selected introns frequently involved in rearrangements and RNA sequencing of 265 genes commonly fused in cancer. The result revealed three genomic alterations: *JAK2* V617F, *NRAS* G13D and an *ETV6*-*ANLN* fusion. The fusion breakpoints occurred in intron 1 of both genes, leading to a putative transcript carrying *ETV6* first exon fused to *ANLN* exons 2 to 25. We confirmed the expression of the fused gene by means of cDNA polymerase chain reaction (PCR) and Sanger sequencing (Fig. 1b).

*ANLN* encodes an actin-bindig protein essential to cytokinesis that is expressed at low levels in most normal tissues [4]. The protein encoded by *ANLN* consists of one,125 amino acids and contains an actin-binding region (amino acids 231 to 676), an Anilin domain (amino acids 799–953), a C-terminal pleckstrin homology domain (amino acids 983–1107) and a nuclear localization region (amino acids 1–230) [4, 5]. The putative protein encoded by the *ETV6*-*ANLN* fusion described here substitutes the first 6 ANLN amino acids (MDPFTE) by the first 11 *ETV6* amino acids (MSETPAQSSIK), resulting in a protein almost identical to *ANLN*, without disrupting its main functional domains. It is unknown at this time if this change disrupts *ANLN* function. On the other hand, this protein lacks all *ETV6* functional domains. We therefore hypothesized that the leukemogenic mechanism operating in this case could be related to the overexpression of the fusion protein, which can have very similar functional characteristics to wild type *ANLN.*

We thus designed two Taqman qPCR assays (Life Technologies) to study the expression level of the fusion gene and also the expression of wild type *ANLN* in healthy volunteer donors and patients with myeloid malignancies. In order to evaluate only the expression of wild type *ANLN,* we designed primers complementary to *ANLN* exon 1 and exon 2. Since the fusion gene lacks *ANLN* exon 1, only wild type *ANLN* was amplified. For the fusion assay, primers were complementary to *ETV6* exon 1 and *ANLN* exon 2. We evaluated the expression of both transcripts in the following magnetic bead selected cell populations: granulocytes (CD66b+) from 20 patients with PMF and 8 healthy volunteers, in CD34+ cells from 10 AML patients and on myeloblasts (CD34+), bone marrow mononuclear cells enriched for megakaryoblasts (CD34-), T cells (CD3+) and granulocytes (CD66b+), from P005. All cell populations had > 95 % purity.

**Fig. 2:**
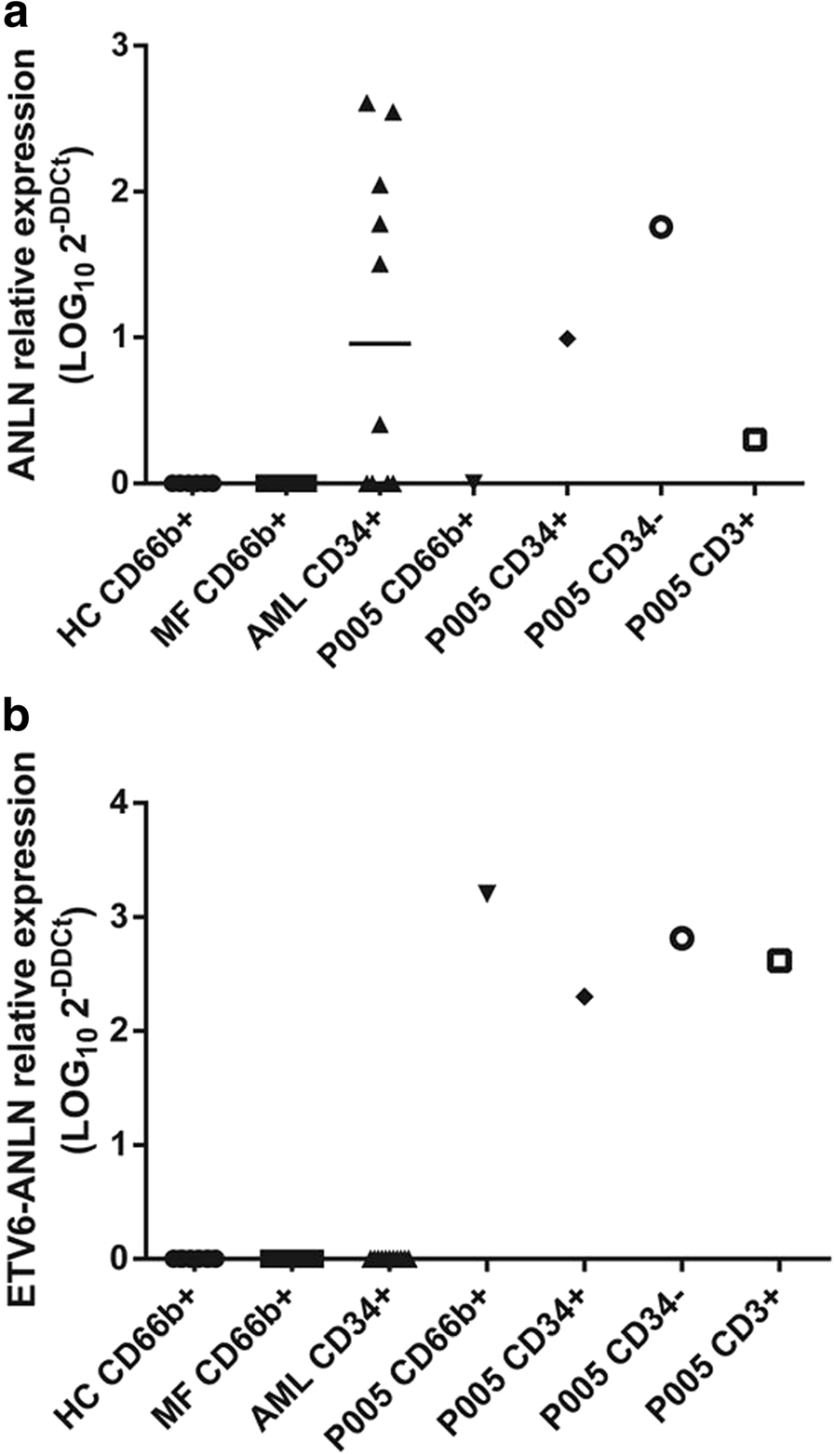
ANLN and ETV6-ANLN expression. **a** qPCR ANLN wild type analysis of cDNA from healthy controls granulocytes (HC CD66b+) *n* = 8, primary myelofibrosis granulocytes (MF CD66b+) *n* = 20, myeloid blasts from AML samples (AML CD34+) *n* = 10, P005 granulocytes, P005 CD34+ blasts, P005 CD34- blasts and P005 CD3+ T lymphocytes. **b** qPCR ETV6-ANLN analysis of cDNA from healthy controls granulocytes (HC CD66b+) *n* = 8, primary myelofibrosis granulocytes (MF CD66b+) *n* = 20, myeloid blasts from AML samples (AML CD34+) *n* = 10, P005 granulocytes, P005 CD34+ blasts, P005 CD34- blasts and P005 CD3+ T lymphocytes. Data was plotted as LOG10 2e-DDCt

Expression of wild type *ANLN* was absent in granulocytes from healthy subjects, PMF patients and P005. On the other hand, *ANLN* expression was present in CD34+ cells from a subset of AML patients and in both CD34+ and CD34- mononuclear populations from P005 (Fig. 2a). These data suggest that wild type ANLN is not expressed in mature granulocytes, but only in CD34+ cells from a subset of AML patients. We did not study the expression of ANLN in CD34+ cells from healthy donors, therefore we cannot rule out ANLN expression in normal CD34+ cells.

On the other hand, the fusion transcript was present in all P005 cell subpopulations (Granulocytes, T lymphocytes, CD34+ blasts and CD34- mononuclear cells), suggesting that the translocation may have occurred in a pluripotent hematopoietic stem cell or an early precursor (Fig. 2b). As expected, no fusion transcript was detected in other individuals.

While more common in lymphoid malignancies, such as acute lymphoblastic leukemia, *ETV6* translocations are uncommon in myeloid neoplasms. In a study of 9,550 patients with myeloid neoplasms, *ETV6* translocations were found in 0.5 % of patients, occurring in only 0.3 % of myeloproliferative neoplasms [1].

Since the fusion gene we identified retains only the first exon of *ETV6*, while maintaining the integrity of the *ANLN* gene, we speculate that the oncogenic mechanism can be related to deregulated *ANLN* expression. Indeed, previous studies have reported that *ANLN* is overexpressed in a variety of human cancers such as lung [6], breast [7] and endometrial cancer [8]. In addition, increased *ANLN* expression has been linked to tumor progression [8], and inhibition of *ANLN* in lung cancer cells decreases cell viability and increases cell size and ploidy, probably secondary to defective cytokinesis [6]. Thus, *ANLN* seems to play an important role in cell division, and increased expression of *ANLN* has been shown to induce DNA synthesis in lung cancer cells [6]. Similar results have been shown in breast cancer, where inhibition of *ANLN* expression abrogates cell proliferation and colony forming ability of breast cancer cell lines. We are not aware of published data about ANLN expression in hematological malignancies, and the precise role of the *ETV6-ANLN* fusion transcript in the pathogenesis of myeloid malignancies with the t(7;12)(p15;p13) translocation still needs to be defined, but we believe that deregulated *ANLN* expression leading to increased cellular proliferation might play a role.

### Conclusions

We have demonstrated that the recurrent AML associated translocation t(7;12)(p15;p13) leads to the formation of the novel fusion gene *ETV6-ANLN* that is expressed in the myeloid and lymphoid lineages. To the best of our knowledge, this is the first report implicating the actin-binding protein *ANLN* in the pathogenesis of AML and other myeloid neoplasms.
